# Accelerated complete human skin architecture restoration after wounding by nanogenerator-driven electrostimulation

**DOI:** 10.1186/s12951-021-01036-7

**Published:** 2021-09-20

**Authors:** Aiping Liu, Yin Long, Jun Li, Long Gu, Aos Karim, Xudong Wang, Angela L. F. Gibson

**Affiliations:** 1 Department of Surgery, University of Wisconsin-Madison, Madison, WI 53792 USA; 2 Department of Materials Science and Engineering, University of Wisconsin-Madison, Madison, WI 53706 USA

**Keywords:** Wound healing, Electrical stimulation, Nanogenerator, Regeneration, Human skin

## Abstract

**Background:**

Electrostimulation (ES) therapy for wound healing is limited in clinical use due to barriers such as cumbersome equipment and intermittent delivery of therapy.

**Methods:**

We adapted a human skin xenograft model that can be used to directly examine the nanogenerator-driven ES (NG-ES) effects on human skin in vivo—an essential translational step toward clinical application of the NG-ES technique for wound healing.

**Results:**

We show that NG-ES leads to rapid wound closure with complete restoration of normal skin architecture within 7 days compared to more than 30 days in the literature. NG-ES accelerates the inflammatory phase of wound healing with more rapid resolution of neutrophils and macrophages and enhances wound bed perfusion with more robust neovascularization.

**Conclusion:**

Our results support the translational evaluation and optimization of the NG-ES technology to deliver convenient, efficient wound healing therapy for use in human wounds.

**Graphic abstract:**

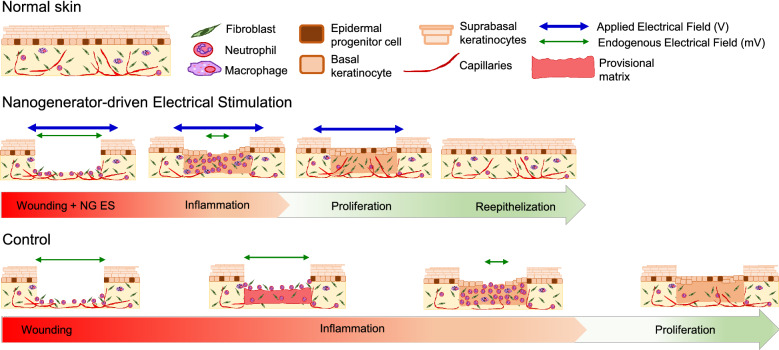

**Supplementary Information:**

The online version contains supplementary material available at 10.1186/s12951-021-01036-7.

## Introduction

Effective, safe, painless and easy-to-use approaches to wound care are highly desired. Despite a robust wound healing product market in excess of $15 billion annually, some currently available therapies have failed to improve outcomes due to a critical lack of preclinical models to inform development of clinically meaningful technologies [1]. Two million patients per year in the US will suffer acute traumatic, surgical, and burn wounds that require advanced wound care [2]. Additionally, chronic wounds affect over 6.5 million people in the US and pose escalating threats to the patient’s quality of life and to the economy, resulting in over $25 billion in annual healthcare expenditures [1, 3–5].

When a wound first occurs, disruption of the epithelial layer leads to the generation of endogenous electric fields. These electric fields are thought to function as cues to direct the migration of epithelial cells for efficient wound healing, although the mechanisms by which electric fields affect healing are poorly understood [6]. Electric fields present an opportunity for a robust wound healing strategy in which the exogenous electric current is harnessed and utilized to decrease prolonged inflammation while improving cellular migration and proliferation [7]. The basic tenet of wound care is to provide an environment that supports orderly proliferation of dermal and epidermal components to facilitate wound closure. Electrostimulation (ES) for wound healing is an attractive adjunct to wound care given that an electric field is essential for directing many cellular processes that lead to orderly healing naturally [8]. Clinical applications of ES treatments for skin wounds are still largely limited due to the suboptimal healing results and inconvenience of implementation. A new promising study recently demonstrated that the nanogenerator (NG)-driven ES (NG-ES) reduced wound healing time from 15 to 3 days on rats with minimal scar [9]. NG is a technology that effectively converts biomechanical energy (e.g. muscle stretching, breathing, and heart beating) into low-frequency and charge-limited electric potential pulses, which would be more favorable for biological systems [10–12].

**Fig. 1:**
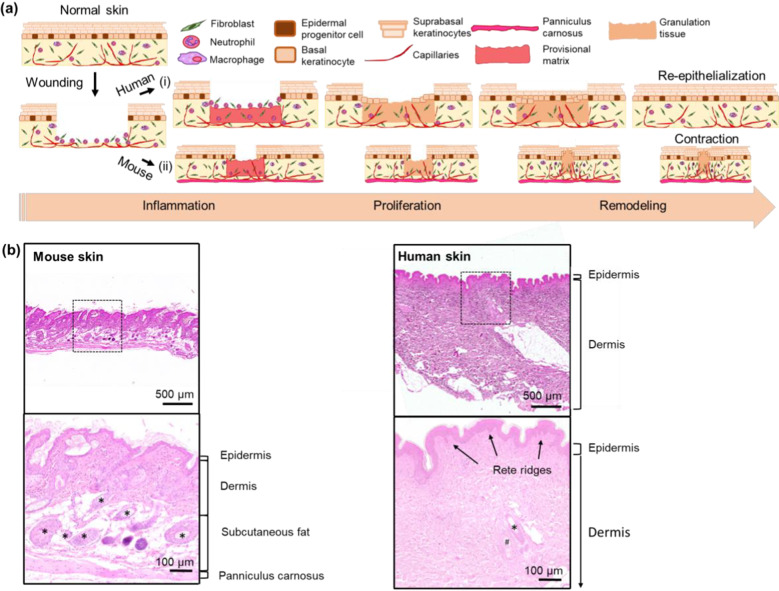
Phases of wound healing and differences between human and rodent skin. **a** An acute wound heals primarily by (i) re-epithelialization in human skin and (ii) contraction in rodent skin. One difference in rodent skin is the presence of the panniculus carnosus, a thin layer of muscle attached to the subcutaneous tissue that serves as the contractile force for wound closure. The overlapping phases of wound healing are similar, with initial inflammation and provisional matrix deposition resulting in the recruitment of neutrophils and macrophages to the wound to assist in clean-up and stimulation of granulation tissue deposition. As the inflammatory phase progresses into the proliferative and remodeling phases, there is a decrease in neutrophils and macrophages. In (i) human skin, a maturation of the granulation tissue extracellular matrix provides a surface for keratinocyte migration and proliferation to occur in a process known as re-epithelialization. In (ii) rodents, the panniculus carnosus and fibroblasts, aid in the contraction of the wound edges and ultimately wound closure by decreasing the overall surface area as the dominant process for wound closure. **b** Anatomical differences in mouse skin and human skin are illustrated by H&E. Mouse skin contains a higher density of hair follicles (denoted by asterisks) that originate in the subcutaneous tissue, lacks eccrine glands and rete ridges, and has very thin dermis compared to human skin. Human skin hair follicles (denoted by asterisk) originate in the dermis, are less numerous and depend on body location. Additionally, human skin has more structures that contribute to re-epithelialization such as eccrine glands (denoted by hash), and rete ridges that also support the strength of epidermal and dermal adhesion. Scale bar = 500 µm low magnification; 100 µm for high-magnification insets

Despite the high efficacy of NG-ES in rodent wound healing, humans have fundamentally distinct wound healing mechanisms and skin architecture, therefore evaluation in rodent models alone is insufficient. Human wounds heal mainly by re-epithelialization, whereas rodents heal primarily by contraction due in large part to the thin muscular layer known as the panniculus carnosus (Fig. 1a), which is absent in humans. Furthermore, there are anatomical differences between mouse and human skin (Fig. 1b) in the thickness of the epidermis and dermis, presence and number of skin appendages that contribute to re-epithelialization and structural stability of skin such as hair follicles, eccrine glands, and rete ridges, and location of these appendages throughout the tissue.

To move this exciting discovery to humans, it is essential to evaluate the impact of ES on the re-epithelialization process of human skin. The use of human skin transplanted onto immunocompromised mice is an effective method used for examining human skin wound healing in vivo [13–15]. Immunohistochemically, the xenografts can maintain morphological, immunological and functional characteristics of human skin even after wounding [13, 16]. Thus, the human skin xenograft model offers a powerful and controllable platform to study cutaneous wound healing in vivo prior to clinical studies in human patients. With this in vivo human wound healing model and a newly designed integrated NG-ES bandage, we studied the impact of NG-ES on the healing processes of re-epithelialization, inflammatory response, and neovascularization at the tissue level. These pre-clinical studies on human tissue provide clinically relevant information to improve our understanding of the effects of NG-ES on human skin and represent a critical step towards the translation of this technology towards their use in patients with wounds.

## Results

### Human skin architecture is preserved in xenograft mouse model

**Fig. 2:**
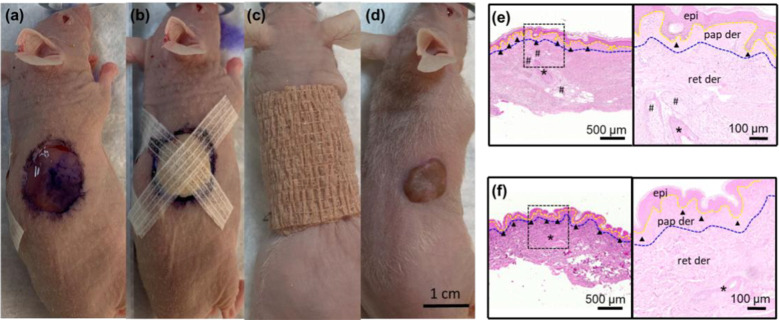
Human skin xenograft preserves normal human skin architecture. **a**–**c** Human skin grafting onto immunocompromised nude mice with scale bar = 1 cm. **d** Human skin xenograft appears viable grossly and is adherent after 8 weeks of engraftment. H&E stains of **e** human skin xenograft after 8 weeks of engraftment and **f** partial-thickness human skin prior to skin grafting. The human skin xenograft preserves the key features of normal human skin including hair follicles (denoted by asterisk), rete ridges (denoted by arrowhead), and eccrine glands (denoted by hash). The epidermis (epi) is separated from the dermis using a yellow dotted line; dermis is composed of papillary dermis (pap der) and reticular dermis (ret der), and their interface is denoted by a blue dotted line. Scale bar = 500 µm low magnification; 100 µm for high-magnification insets

Figure 2a–d demonstrates a typical procedure of an immunocompromised mouse undergoing a partial-thickness ~ 1.2-cm diameter human skin xenograft. After 8 weeks of engraftment, the transplanted human skin appears grossly viable and adherent to the wound bed (Fig. 2d). The overall area of the human skin xenograft was reduced to about 50% of its original size owing to the tension of the surrounding mouse skin and panniculus carnosus, illustrating the strong contractile forces in murine wound closure that is distinct from human wound healing. Eight weeks after grafting, the human skin xenografts (Fig. 2e) possess key histologic features of normal human skin including hair follicles, rete ridges, and eccrine glands and are similar in appearance and density to the human skin prior to grafting onto the mouse (Fig. 2f). The epidermis is fully differentiated, and its thickness is comparable to its original grafted skin. The dermis retains the papillary and reticular dermis although the reticular dermal layer is thicker compared to the original grafted skin likely related to the contraction of the mouse skin around the xenograft after initial healing as noted above. The gross and histologic normal appearance of the human skin 8 weeks after grafting signals the readiness of the xenograft for wound healing experimentation.

### Self-powered wearable ES bandage

**Fig. 3:**
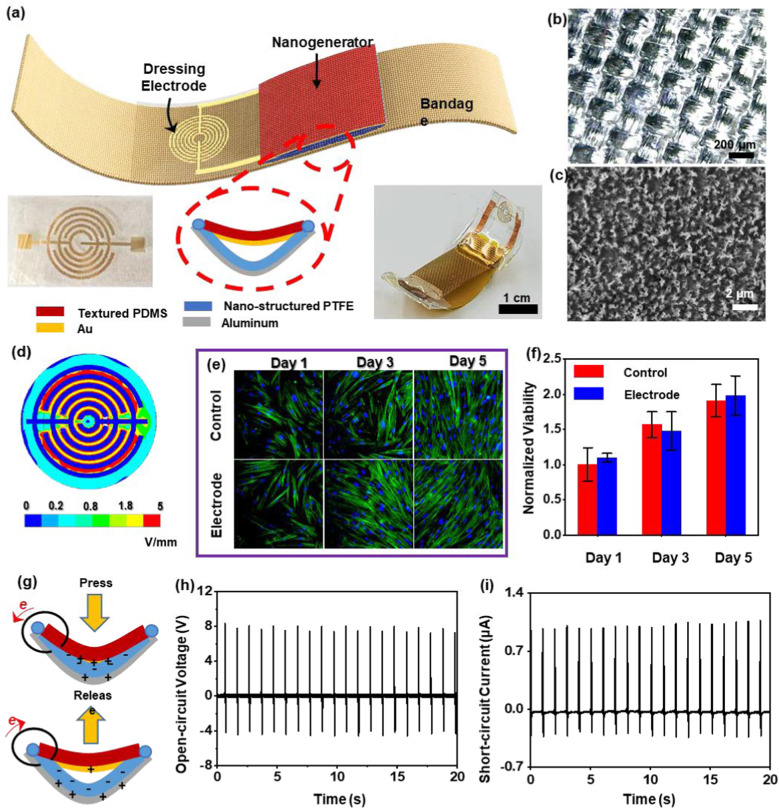
NG-ES bandage development. **a** Schematics of NG-ES bandage consisting of a triboelectric nanogenerator (TENG), a pair of dressing electrodes, and a bandage base. Left inset is a digital image of the dressing electrodes. Middle inset is a schematic of arc-shaped NG. Right inset is a digital image of a NG-ES bandage. The scale bar = 1 cm. **b** SEM image of micro-structured PDMS surface replicating textile mat. **c** SEM image of dense nanowire arrays on the PTFE surface. **d** FEA simulation of electric field distribution in between the dressing electrodes when a 1 V electric potential is applied. **e** Fluorescence microscope image of human fibroblast cells cultured on dressing electrodes surface and normal cell plate (control). **f** Quantitative cell viability as a function of time. **g** Schematics of working mechanism of arc-shaped NG under compression showing the flow of electrons “e”. **h** Open-circuit voltage and **i** short-circuit current output of the as-prepared NG compressed at a frequency of 1 Hz by a computer-controlled actuator

A wearable ES bandage was fabricated to provide self-powered ES for accelerated skin wound healing on mice. It consisted of a pair of dressing electrodes and a stretchable NG that is integrated with a soft bandage (Fig. 3a). The gold dressing electrodes were fabricated by electron beam evaporation on a transparent polyethylene terephthalate (PET) membrane. They were designed as a circular interdigitated configuration, which provides a uniformly high-intensity electric field under the electrode-covered area when an electric potential is applied. To emulate skin accommodating strain, the NG was designed with a unique arc shape, enabling moderate stretchability (~ 20%). This arc-shaped NG was made of gold (Au)-coated polydimethylsiloxane (PDMS) film and aluminum (Al)-covered polytetrafluoroethylene (PTFE) film. It operates under the mechanism of triboelectric effect, and outputs alternative current (AC) electric signals upon the contact and separation of the PDMS and PTFE films. The PDMS layer has skin-mimicking mechanical properties that conforms to the mouse skin to sense its gentle movements. PTFE has strong electrification when interfaced with PDMS. Knitted microstructure was created on the PDMS film by replicating the texture surface to improve the film’s strain sensitivity and stretchable (Fig. 3b). Inductively coupled plasma (ICP) treatment was used to fabricate nanowire arrays on the surface of PTFE. The nanowires had average lengths around 1–2 μm with diameters less than 200 nm, which significantly improved the surface roughness, and thus enhanced the triboelectric output (Fig. 3c). The elemental analysis of PTFE was performed using energy-dispersive X-ray spectroscopy (Additional file 2: Figure S1).

A finite element analysis (FEA) simulation was first implemented to examine the electric field distribution from the dressing electrodes. As shown in Fig. 3d, when 1 V electric potential was applied, a high-intensity field of ~ 5 V mm^−1^ would be concentrated between the electrode fingers, while only a weak field (< 0.5 V mm^−1^) was observed outside of the electrode-covered area. This distribution of the electric field is highly desirable for wound stimulation, as the wound covered by the dressing electrodes receives strong electrostimulations, while the tissue surrounding it is minimally affected by the marginal field. Additionally, the concentric design of the electrodes ensures that the applied electric field will evenly cover all directions of wound healing. Since the dressing electrode directly interfaces with the wound, its biocompatibility is of great importance. The biocompatibility was confirmed by culturing human fibroblast cells (HFCs) on the electrode surface. Immunofluorescence staining was performed over a five-day period to examine the cell attachment, morphology, and proliferation. As shown in Fig. 3e, cells attached to the surface after 1 day in culture. They all exhibited normal behavior and reached a higher density with typical spindle morphology over time in culture. The cell morphology, distribution, and densities did not show any significant dissimilarity between the electrode group and control group. Quantitative analysis obtained by 3-(4,5-dimethylthiazol-2-yl)-2,5-diphenyltetrazolium bromide (MTT) assay revealed that the cell viabilities of electrode and control groups have close values without significant differences (Fig. 3f), supporting the nontoxicity of the dressing electrode in contact with cells. NG performance was studied under computer generated gentle and pulsatile forces (peak value of 3 N) at a frequency of 1 Hz, mimicking biomotions applied to the NG by the programmed actuator. The contact and separation between tribo-active Au and PTFE (Fig. 3g) produced AC electric pulses, which were transferred to the connected dressing electrode and eventually delivered to targeted skin wounds. The NG generated a high open-circuit voltage of ~ 8.0 V (Fig. 3h) and a short-circuit current of ~ 1.0 μA (Fig. 3i). These sufficiently large outputs in response to weak stimuli was largely attributed to the surface modification of triboelectric layers. Moreover, stretching of NG could also generate electrical output. As shown in Additional file 2: Figure S2, a weaker voltage output of ~ 4.2 V and current output of ~ 0.34 μA were obtained when the NG was stretched at 20% strain.

### Fibroblast alignment in response to ES

**Fig. 4:**
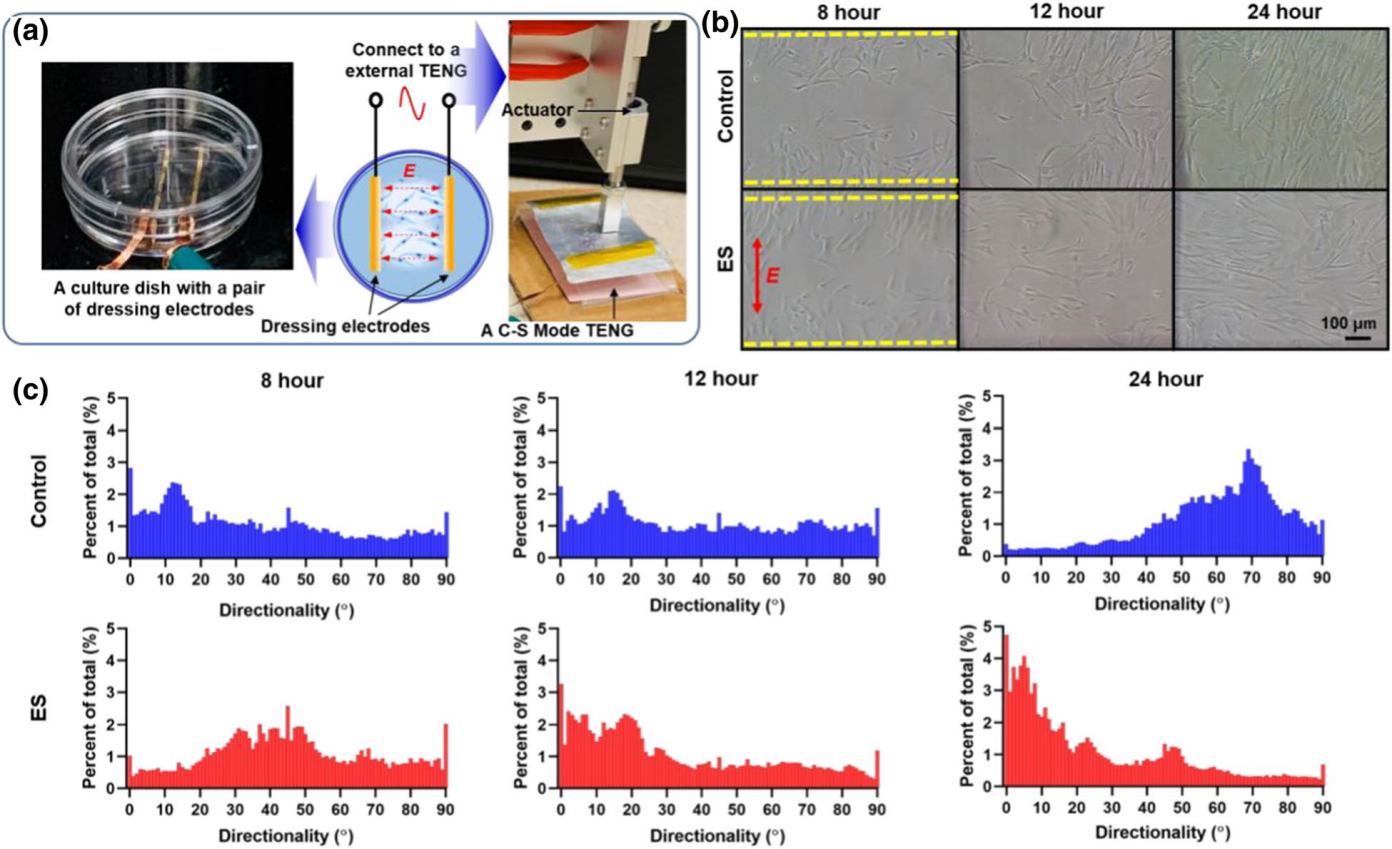
ES modulates the directionality of human fibroblasts. **a** Custom cell culture dish with embedded Au electrodes connected to an actuator simulating the in vivo electric field for ES treated human fibroblasts. **b** Photographs of a representative region of monolayers fibroblasts in cell culture over time. Dashed yellow lines indicate the edge of initial wounded region. **c** Directionality histograms of the orientation of the fibroblast long axis without and with ES treatment for 8, 12 and 24 h. 90° aligns parallel to the direction of the electric field, indicated by the red arrow in **b**. Scale bar = 100 µm

Human fibroblasts are the main cell type in the dermis and are responsible for synthesis, deposition and remodeling of the extracellular matrix that allows for epithelial cell migration to facilitate wound closure. We have previously shown that rat fibroblasts treated with ES demonstrated alignment in response to the ES electric field [9]. As the first step toward human-relevant models, we sought to investigate if human dermal fibroblast alignment would be altered with ES treatment in a wounded environment using a monolayer scratch assay. Monolayer human dermal fibroblasts arrested for cell proliferation with mitomycin-c were grown in culture dishes fabricated with parallel electrodes hooked up to an actuator to simulate NG (Fig. 4a) and treated with ES for up to 24 h. We found that the mitosis-arrested fibroblasts migrated to populate the wound area over time in treated and control scratch assays (Fig. 4b). Under the influence of ES, the long axis of cells preferentially aligned perpendicular to the electric field, as has been reported previously [17]. This tendency was not found in the non-treated fibroblasts. We quantified the cellular directionality in the scratch wound (gap) using Image J [18] to identify the long axis of fibroblasts (ES or control) with the applied electrode field and the direction of the gap to be closed (Fig. 4c). The parallel pair of electrodes was defined as 0° and the scratch wound was created parallel to the electrodes. Directionality histograms confirmed that most cells under ES altered their long axis from 45° to the electric field at 8 h of continuous stimulation to less than 10° after 12 and 24 h of ES, aligning nearly perpendicular to the electric field. Without ES, there was random directionality of most fibroblasts at 8 h which aligned progressively perpendicular to the initial scratch wound at 24 h. These findings highlight the importance of electric field directionality when designing electrodes for various wound shapes and informed the redesign of the electrode for the in vivo studies.

### Accelerated complete human skin wound healing

**Fig. 5:**
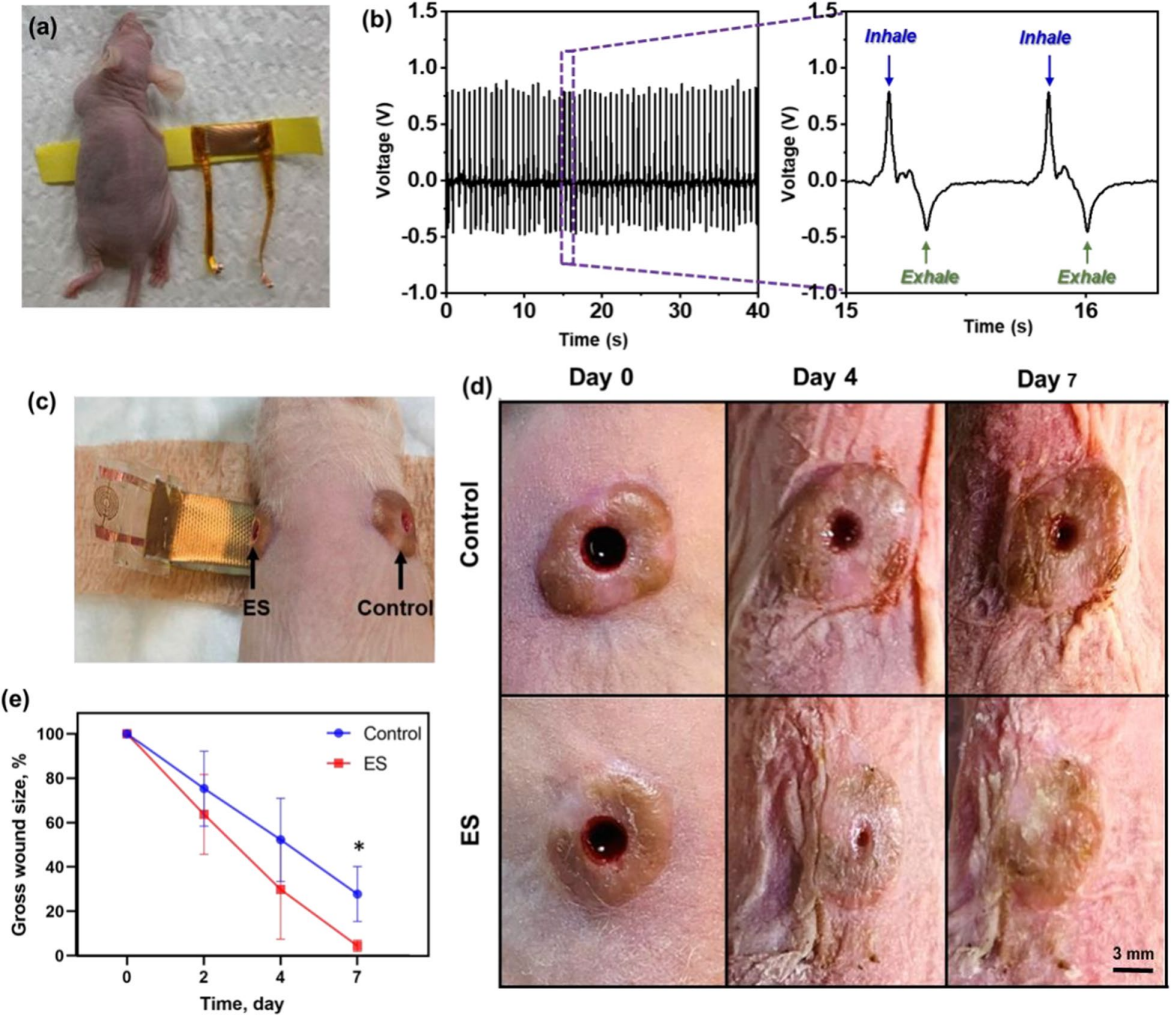
NG-ES accelerated wound healing in human skin xenografts on mice.** a** A mouse wearing an ES bandage for output testing. **b** Voltage outputs obtained from mouse respirations (left) and enlarged voltage output profile showing the peak width and shape in response to inhale and exhale (right). **c** Demonstration of an ES bandage application on the excisional wounds created on human skin xenografts. **d** Representative gross photograph of wound healing and **e** gross wound size in ES treated or non-treated control wounds over 7 days treatment (n = 4 per group). The scale bar = 3 mm. *p < 0.05

While our previous studies demonstrated the beneficial effects of NG-ES on wound healing in rat wounds, there are vast differences in skin anatomy and wound healing between rodents and humans as detailed in Fig. 1. To confirm that the beneficial wound healing effects of NG-ES persist in human skin, we evaluated our ES bandage on full thickness excisional wounds in human skin xenografts on athymic mice (Fig. 5). This human skin xenograft mouse model maintains all the local anatomy of human skin, including hair follicles, eccrine glands, capillaries, resident immune cells, and extracellular matrix to allow an in-depth study of the local tissue architectural effects of ES on human skin, while providing the systemic immune response from the mouse to recapitulate the entire human wound healing processes. Prior to any interventional studies, the voltage outputs from the NG were evaluated on the mouse skin by wrapping the bandage around the torso of the mouse (Fig. 5a). The peak to peak voltage was larger than 1 V directly driven by mouse respirations while under anesthesia (Fig. 5b; Additional file 1: Video S1), which is within the physiological range of electric potential generated endogenously [19].

Initially, the feasibility of using the NG-ES bandage set up was tested on full thickness excisional wounds in mouse skin (Additional file 2: Figure S3a) and demonstrated more rapid wound closure on day 7 in ES treated wounds compared to non-treated controls as determined by gross evaluation (Additional file 2: Figure S3b). Histologic confirmation of wound closure on day 7 samples revealed complete wound closure in the NG-ES treated wounds (Additional file 2: Figure S3c). Given the differences in rodent and human skin, and the desire for rapid translation into human studies, we chose to continue the remainder of our evaluation in our human skin xenograft model. Fully vascularized bilateral flank human skin xenografts were wounded using a punch biopsy to create uniform 3-mm diameter full thickness excisional wounds. Both wounds were wrapped with the ES bandage around the torso, and each was covered by a set of dressing electrodes, where one was connected to the NG component (ES treatment site), and the other one was without NG connection (control site) (Fig. 5c; Additional file 2: Figure S3a). NG-ES was delivered continuously for 1 week. Wound healing, assessed visually at various time points, demonstrated that ES-treated wound size dramatically decreased in size by day 4, compared to the control (Fig. 5d). Quantification of the NG-ES treated wounds revealed that in the presence of continuous NG-driven ES, the wound area reduced rapidly and reached complete closure on day 7, whereas without ES, the wound size slowly reduced to ~ 30% of the original size on day 7 (Fig. 5e).

**Fig. 6:**
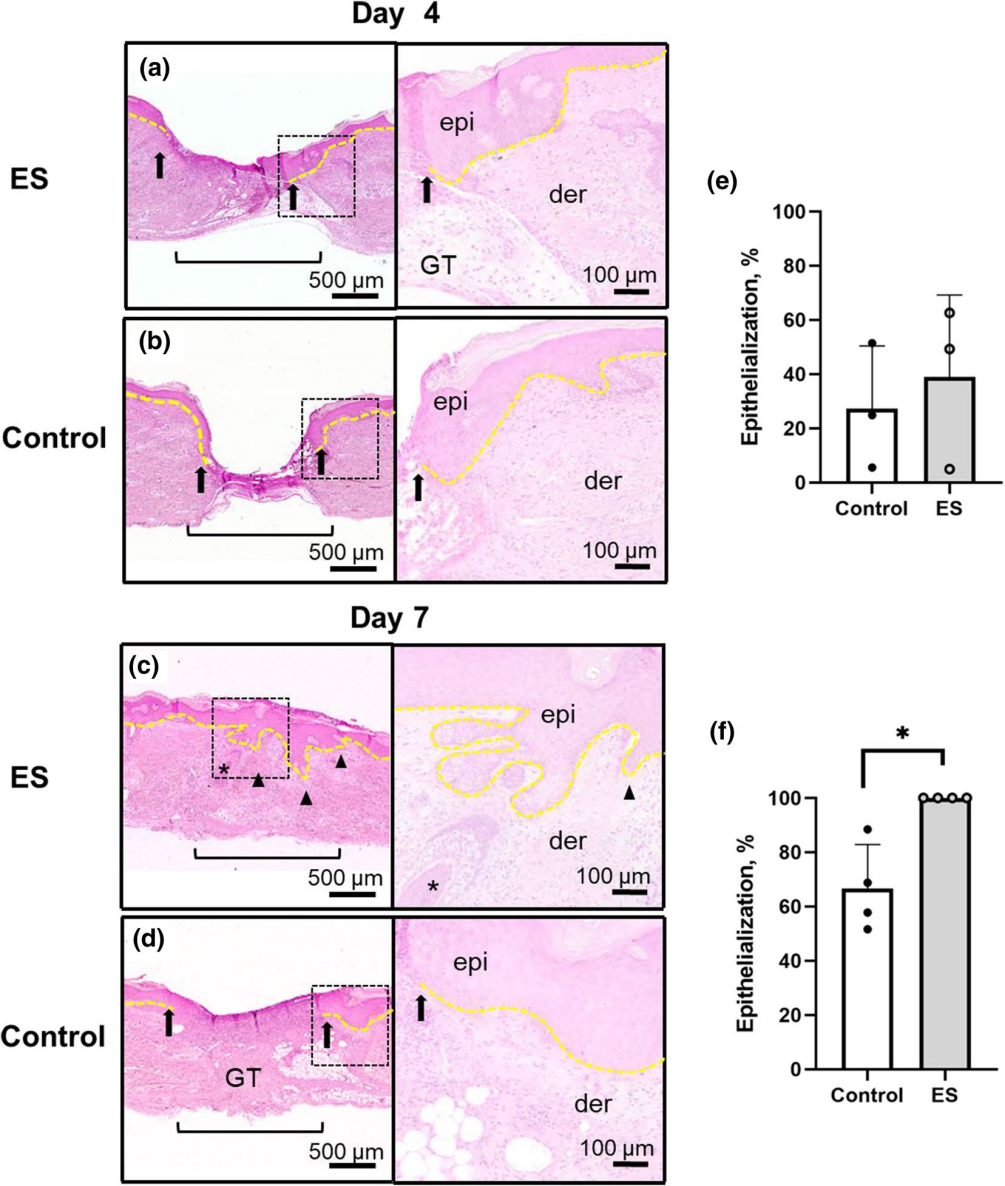
NG-ES facilitated restoration of normal tissue architecture. Representative H&E stained sections of wound region (denoted by black bracket) after (**a**, **b**) 4 and (**c**, **d**) 7 days with or without NG-ES treatment. Leading edge of the neo-epidermis is denoted by black arrows and entire basal epidermal layer is denoted by yellow dotted line. (**c** and inset) Day 7 of NG-ES treated wound demonstrating complete re-epithelialization (entire dotted yellow line) in wound region and reconstitution of normal architecture including hair follicle (denoted by asterisk) and rete ridges (denoted by arrowheads) also seen in low magnification. Scale bar = 500 µm low magnification; 100 µm for high-magnification insets. GT, granulation tissue; epi, epidermis; der, dermis. **g**, **f** Percentage of epithelialization with or without NG-ES treatment at 4 days (n = 3 per group) and 7 days (n = 4 per group) post-injury. *p < 0.05

Comparison of hematoxylin and eosin (H&E)-stained sections of treated and control human skin wounds shows enhanced re-epithelialization and complete wound closure with normalization of human skin architecture in the wound bed after 7 days of ES treatment (Fig. 6). On day 4, granulation tissue has filled the wound bed in the ES treated tissue (Fig. 6a); whereas in the control wound bed there is a lack of granulation tissue at this earlier time point (Fig. 6b). Proliferating epidermal cells are seen migrating laterally across the granulation tissue matrix to close the wound in the ES treated cells (Fig. 6a yellow dotted line and arrows). Granulation tissue provides the surface to support epidermal cell migration, therefore the lack of granulation tissue in the control wounds likely impeded the closure of the wound bed by re-epithelialization. This finding supports ES-accelerated deposition of granulation tissue by fibroblasts as a contributing factor in the early re-epithelialization in ES treated wounds compared to control. By day 7, the wound area has completely re-epithelialized with reconstitution of skin appendages such as hair follicles with full differentiation of the epidermal layer in the ES-treated wounds (Fig. 6c). In contrast, the control wound lacks complete re-epithelialization over a large amount of granulation tissue and inflammatory cell infiltrate (Fig. 6d). Importantly, in ES treated wounds there was reconstitution of rete ridges—an important skin architectural characteristic lacking in rodent skin that enhances the adhesion of the epidermal and dermal layers. Notably, rete ridges are absent in scar tissue. Semi-quantification of percentage of re-epithelialization of the ES-treated and control wounds at day 4 and 7 (Fig. 6e, f) further support that NG-ES can effectively facilitate human skin wound healing via re-epithelialization.

### ES modulates cellular behavior in human skin

**Fig. 7:**
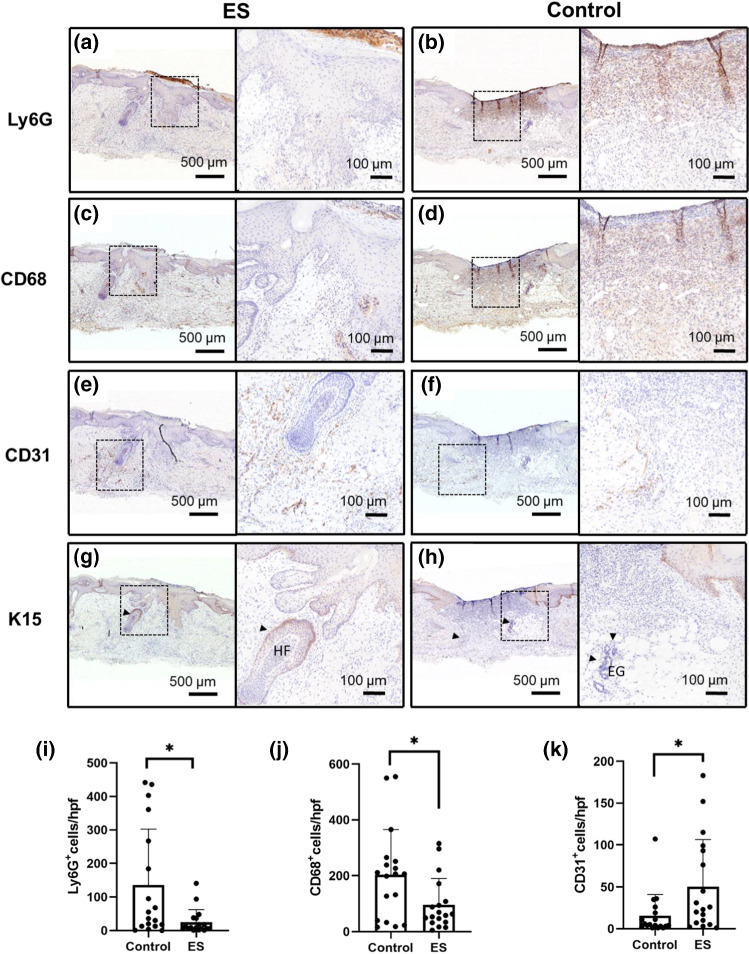
NG-driven ES modulated cells that are critical for wound healing. After 7 days of ES treated or non-treated wounds in the human skin xenografts on mice, (**a**, **b**) neutrophils, shown as brown Ly6G-positive cells, (**c**, **d**) macrophages, shown as brown CD68-positive cells, (**e**, **f**) endothelial cells, shown as brown CD31-positive cells, and (**g**, **h**) epidermal progenitor cells, shown as brown K15-positive cells were evaluated. Arrowheads indicate skin appendages such as the hair follicles (HF) and eccrine glands (EG) in the K15 stained samples. Representative images of replicate experimental animals. Scale bar = 500 µm low magnification; 100 µm for high-magnification insets. **i**–**k** Semi-quantification of Ly6G, CD68 and CD31 positive cells in 6 regions of interests at 400X magnification in the wound beds with and without 7 days of NG-ES treatment (n = 3 per group), respectively. *p < 0.05

Wound healing is a highly orchestrated process involving many cellular players progressing through the various overlapping phases to achieve wound closure as illustrated in Fig. 1. To understand the wound healing processes affected by NG-ES that contributed to the rapid and normal wound healing we observed in human skin xenografts, we used immunohistochemical staining to identify cellular markers on serial tissue sections (Fig. 7). The inflammatory phase of wound healing is characterized by the infiltration of leukocytes, first neutrophils and then macrophages, to clean up wound debris and prevent infection. However, prolonged inflammation contributes to aberrant chronic wound healing [20]. To understand the contribution of ES on the inflammatory response, we performed immunohistochemistry for neutrophils and macrophages on tissues harvested after 4 and 7 days of ES to assess the dynamics of the inflammatory cell response and resolution in the wound healing process. We found that on day 4, the neutrophils were highly concentrated in the granulation tissue and surrounding dermis in the ES-treated wounds and in the dermis of the control wound (Additional file 2: Figure S4a, b, i). By day 7, neutrophils were greatly reduced in the ES-treated wounds to a baseline level whereas an accumulation and persistence of neutrophils existed in the control wounds (Fig. 7a, b, i). We found that macrophages followed a similar temporal pattern of presence and resolution to that of neutrophils at day 4 (Additional file 2: Figure S4c, d, j) and day 7 (Fig. 7c, d, j) in ES-treated and control wounds. These findings illustrate that ES treated wounds have a faster resolution of the inflammatory response that coincides with normal wound closure.

Angiogenesis is critical for nutrient delivery and toxin removal to support cell proliferation and granulation tissue formation during wound healing. Endothelial cells, participating in angiogenesis accumulate near the edge of both ES-treated and control wounds by day 4 (Additional file 2: Figure S4e, f, k). By day 7, the presence of endothelial cells migrating into the wound in the ES-treated tissue was enhanced compared to the control tissue (Fig. 7e, f, k), indicating that our NG-driven ES stimulates angiogenesis in wound healing, as has been shown in other ES studies [21].

The regenerative capacity of the skin relies on the local populations of epidermal stem cells in the interfollicular epidermis and the skin appendages, which participate in re-epithelialization in response to injury. To understand the effects of ES on epithelial regeneration, we stained for keratin 15 (K15), a marker for epidermal progenitor cells [22]. In contrast to the high and localized expression in the intact basal layer outside the wound margin, K15 was expressed at a low intensity and dispersed in the advancing edge of neo-epidermis in both control and ES-treated wounds on day 4 and day 7 (Additional file 2: Figure S4g, h; Fig. 7g, h). This down regulation of K15 at the neo-epidermis is associated with wounding in response to transforming growth factor beta (TGF-β) [9, 23, 24], a multifunctional cytokine we have previously found to be upregulated by NG-ES in rodents [9]. Interestingly, we found that K15 expression in skin appendages such as the hair follicles and eccrine glands (Fig. 7g, h arrowhead), where other sources of stem cells reside and participate in the repair of the epidermis after injury, was increased in the ES-treated wounds compared to the control wounds. This finding suggests that ES preferentially stimulates epidermal progenitor cells in the skin appendages rather than in the neo-epidermis to facilitate restoration of tissue integrity via these dermal structures.

### Conclusions and discussion

In this work, we demonstrate significantly enhanced healing of acute human skin wounds treated with NG-driven ES. This self-powered ES was made possible by an ES bandage that includes a flexible microstructured NG effectively converting biomechanical energy from respiration to electric pulses. The human skin xenograft model on mice clearly revealed that under NG-ES treatment, a 3 mm full thickness excisional wound closed within seven days with complete return of normal human skin architecture; whereas similar human wounds (4 mm) typically required > 30 days to achieve full closure [25]. Tissue level studies showed that NG-ES led to an effective and more rapid inflammatory resolution, more robust neovascularization, and enhanced stimulation of epidermal progenitor cells. Together, these cellular behaviors directed by NG-ES facilitated complete wound closure with a rapid return to baseline upon closure.

As a potential surface-contact medical device, the biosafety and biocompatibility of the NG-ES device warrants additional considerations. The surface of the NG and the electrode in contact with the skin was coated with a thin layer of PDMS. PDMS is a well-known biocompatible and bio-stable material [26], which is routinely used as a biomedical implant material and in biomedical research [27]. In the context of skin, PDMS has not been shown to be a skin irritant or a skin sensitizer [26]. In our in vitro study on human fibroblasts, we found no evidence that the thin layer of PDMS affected cellular behaviors such as cell morphology, proliferation and migration (to close the gap in the scratch assay). The mouse skin that directly contacted the NG or electrode did not show any signs of irritation, inflammation, infection, or necrosis. We did not observe any changes in animal weights, breathing, sleeping, eating rituals as a result of NG-ES treatment, nor did any of the mice display hunched posture, fur loss, reduced mobility, or tachypnea that would lead us to believe that there were deleterious systemic effects of the NG. The cytotoxicity and biocompatibility assessments in our in vitro and in vivo animal studies enhance the translational potential of NG-ES to patients.

Another concern of NG-ES is that unlike the endogenous electric stimulation, NG-ES does not terminate when the epithelium is fully re-epithelized, which may lead to hyper-proliferation of the epithelial layer during reepithelization. Normal wound healing involves cell contact-inhibition signaling in the cell proliferation, growth and differentiation [28]. Keratinocytes activated by wounding migrate to cover the wound and proliferate to form a dense hyperproliferative epithelium feeding the epithelial tongue that extends from both ends of the wound [29]. When the two epithelial tongues fuse at the middle of the wound, contact inhibition stops the migratory process of keratinocytes, and they undergo programmed terminal differentiation to result in epidermal stratification. ES has been shown to enhance both migration and proliferation of keratinocyte in the process of epithelization [30, 31]. To address the concern whether NG-ES leads to excessive-epithelization, we quantified the thickness of the newly regenerated epidermis at the wound site between the NG-ES and the control groups (Additional file 2: Figure S5). We did not observe any difference in the thickness of the neo-epidermis between the two groups, suggesting that ES did not alter the normal mechanisms of re-epithelialization.

While the use of the human skin xenograft model is as close to studying NG-ES treatment in human skin wounds as possible without using human subjects, we recognize that the lack of a human immune response is a limitation in our studies. Our findings are crucial in the successful translation of this NG-ES technology for human wound healing clinical trials, and this model ideally serves a necessary step to validate the effects of NG-ES in human skin prior to human subject testing. The next steps include optimization of the NG and bandage design to harness the biomechanical energy at various human body sites to allow future application of this exciting ES-facilitated wound healing phenomenon in humans. The fundamental tissue-level understanding of cellular behavior reported here supports the continued investigation of NG-ES as a promising strategy in treating more challenging skin wounds, such as infected, chronic and burn wounds.

## Materials and methods

### Study design

The primary objective of this study was to determine whether ES driven by body motions through a nanogenerator could accelerate normal wound healing in human skin. To study wound healing, mice with human skin xenografts were wounded and treated with NG-ES or electrode without connection to the NG as a control. Secondary objectives were to identify what tissue level processes participated in the accelerated healing using histology and immunohistochemistry in situ*.* Researchers could not be blinded to the treatment conditions given the connection or lack of connection of the electrodes to the nanogenerator on the wounds. Each mouse served as its own control with bilateral flank xenograft wounds. All wounding experiments were performed with 14–15 week old male mice (n = 3–4 per group as indicated in the figure legends).

### Fabrication of NG-based ES bandage

A PTFE (CS Hyde Co.) film (2.7 cm × 1.0 cm × 75 μm) film was used as the triboelectric active layer, and a pieces of Cu foil (2.7 cm × 1.0 cm × 25 μm) were attached to back side of PTFE substrate as one electrode. Nanowires were created on the surface of PTFE by the inductively coupled plasma (ICP) (Plasma Therm 790 ICP/RIE Etcher) to further enhance the contact area and surface charge density. In a typical ICP process, Ar, O_2_ and CF_4_ gases were introduced into the ICP chamber with a flow rate of 15.0, 10.0, and 30.0 sccm, respectively. The first power source of 400 W was used to generate a large density of plasma and the other power of 100 W was used to accelerate the plasma ions. The PTFE film was etched for 120 s with a pressure of 10 mTorr. A thin Kapton film (CS Hyde Co.) (2.7 cm × 1.0 cm × 50 μm) was used as package layer to cover the Cu foil. Microstructured PDMS film (2.5 cm × 1.0 cm × 100 μm) was made by replicating the surface of non-stick Teflon heat press mat (Selizo). 100 nm gold was deposited through slow E-beam Evaporation (CHA-600) on the PDMS surface as both another electrode and triboelectric layer. The gold deposited PDMS film was stretched to the same length as Cu/PTFE film. After fixing the two ends of both films by epoxy (Devcon), the stretching stress was released on PDMS, and an arc shape was formed due to unmatched length. Working electrodes (Au 50 nm and Cr 10 nm) for electrical stimulation were deposited on the PET substrate by E-beam Evaporation through customized shadow masks. The same working electrodes were used in control experiment without connecting the NG electrodes.

### Electrical characterization of NG-based ES bandage

The compressing/stretching of arc-shaped NG was driven by a computer-controlled actuator (LinMot) at a frequency of 1 Hz. The voltage outputs of NG were measured by connecting probes of a multimeter (DMM 6500, Keithley) to the working electrodes. The short-circuit current was measured by an electrometer (Keithley 6514) connected with LabVIEW system in computer. For animal studies, the voltage outputs of NG were measured by the multimeter when the mouse was under anesthesia.

### Cell morphology and immunofluorescence staining for biocompatibility study

After human fibroblast cells [32] were cultured on the top of the working electrode film in the culture plate, the cytoskeleton and nucleus were stained with Flash Phalloidin Green 488 (BioLegend) and blue fluorescent Hoechst (352/461 nm) (Thermo Fisher Scientific), respectively. The Flash Phalloidin Green 488 was reconstituted with 1.5 ml of methanol to make 300-unit stock solution. The samples were fixed with 2–4% formaldehyde for 15 min and then rinsed three times with prewarmed PBS. The samples were incubated with Flash Phalloidin Green 488 (diluting 300 µl stock solution 1:50 in 1× PBS) and Hoechst (1 µg ml^−1^) for 30 min at 37 °C. After staining, the cells were rinsed with prewarmed buffer for three times and imaged samples using a Nikon A1RS high definition (HD) Confocal microscope.

### Cell cytotoxicity

The cell cytotoxicity of package NG and electrodes was assessed with the 3-(4,5-dimethylthiazol-2-yl)-2,5-diphenyl-2*H*-tetrazolium bromide (MTT) assay using human embryonic kidney 293 (HEK293) cells (American Type Culture Collection). Generally, the cells were seeded in a 96-well plate (5 × 10^3^ cells/well) and incubated for 24 h at 37 °C and 5% CO_2_, then working electrode materials were added to the wells. After incubation for 20, 68 h, and 116 h, 20 μl MTT solution was added to each well and incubated for another 4 h. The absorbance of each well was measured at 490 nm using ClarioStar Plate Reader. Nontreated cells were used as control, and the relative cell viability was expressed as (Abssample − Absblank)/(Abscontrol − Absblank) × 100%, where Absblank is the optical density of the wells without any cells.

### Xenograft mice and electrical stimulation treatment

To test the efficacy of NG-driven ES on human skin wounds, we used an established xenograft mouse model of human skin wound healing [15]. The de-identified human skin tissues were obtained through an Institutional Review Board exempt protocol in accordance with laws and regulations of the University of Wisconsin-Madison School of Medicine and Public Health. All procedures on mice were approved by the University of Wisconsin Institutional Animal Care and Use Committee and the Research Animal Resource and Compliance office (M006243). Briefly, immunocompromised nude mice (6–7 weeks old) were purchased from Jackson laboratory (Bar Harbor, ME, USA). After mice were adequately anesthesia under isoflurane, full thickness mouse skin (1.4 cm in diameter) was removed from two graft beds, one on each flank (Fig. 2a). The partial-thickness human skin (1.2 cm in diameter, ~ 1.0 mm thickness) procured from elective surgeries was placed onto each mouse wound bed, and secured with 3 Steri-Strips™ (3M) with the mouse skin margin splinted 2-mm away from the border of the xenograft (Fig. 2b). The xenografts were covered with Cuticerin^®^ (Smith and Nephew) and bandaged using 1 inch wide CoFlex (Andover) until week 4 post grafting (Fig. 2c). The Steri-Strips were removed from the grafts on week 2.

Eight weeks after grafting to allow for human skin engraftment and normalization on the mouse wound bed prior to wounding (Fig. 2d), full thickness excisional wounds were created in the human skin graft using a 3-mm diameter biopsy punch (Fig. 5c, d). Mice were bandaged around the torso with one wound treated with NG-driven ES and the other without treatment as the control. Wounds were observed grossly throughout the healing process. The wound closure was quantified as wound area over time normalized by its own initial wound area (on day 0).

### Histology and image analysis

To understand the impact of ES on wound healing processes at the tissue level, human skin grafts were harvested on day 4 or 7 and processed for H&E and various immunohistochemistry (IHC) stains. H&E staining was performed to evaluate re-epithelialization and tissue architecture. Epidermal progenitor cells (K15, ab111448, 1:1000), neutrophils (Lymphocyte antigen 6 complex locus G6D or Ly6G, BioLegend 127602, 1:500), macrophages (cluster of differentiation 68 or CD68, ab125212, 1:500) and endothelial cells (cluster of differentiation 31 or CD31, ab76533, 1:400) were stained to identify critical cellular processes in wound healing. Tissue sections were viewed using a Nikon Ti-S inverted microscope and digital images were captured with Nikon DS Ri2 cooled color camera, X-Cite 120LED BOOST System lamp from Excelitas, and Nikon Imaging Software, NIS Elements (Nikon, Tokyo, Japan). Bright field images were taken at 40–400× magnification.

Quantification of the degree of epithelialization on the H&E slides was performed with NIS Element Imaging Software (Nikon) to trace the distance between the advancing edges of the neo-epidermis of each H&E stained sample. The percentage of epithelialization was calculated from the ratio of the distance and its original wound size (3 mm). The quantification of Ly6G, CD68 and CD31 positive cells was performed with Fiji [33–35]. Six regions of interests were randomly selected at 400× magnification within the wounded region from each tissue section. The color deconvolution tool in Fiji was used to extract the target staining colors. Thresholds were then set for the images to identify the area with positive staining and nuclei, respectively. The positive cell numbers were counted as the number of nuclei that reside in the positive staining areas by using Image Calculator and Particle tools in Fiji.

### Scratch assay for directed cell migration

To understand the impact of ES on the migration of fibroblasts in wound healing, a widely used in vitro wound healing assay (e.g., scratch assay) was performed as previous described [36]. Briefly, human fibroblasts cultured in a p35 tissue culture dish (Corning) with a pair of parallel electrodes adhered to the bottom of the plate (Fig. 4a). When they reached to 90–100% confluence, the cells were pretreated with 10 µg ml^−1^ of mitomycin C (Sigma Aldrich) for one hour to prevent the confounding influence of cell proliferation. After a PBS rinse, a scratch (~ 0.44 to 0.5 mm width wound) was performed using a 200 µl sterile pipette tip perpendicular to the bottom of the dish. A subset of fibroblasts was then treated with NG-ES for 24 h. The gold electrodes were deposited through shadow mask by slow E-beam evaporation (CHA-600). The electrodes were directly deposited on the surface of culture dish with a thickness around 100 nm, which were followingly packaged by a thin layer of PDMS (~ 10 µm). Images of the same wounding sites were taken at various time points after the wounding under a microscope (Nikon). The orientation of cells in relation to the wound region was quantified using the directionality histogram in Image J [18].

### Statistical analysis

All statistical analyses were performed using Graphpad Prism version 8 for Windows (Graphpad, La Jolla, CA, USA). Quantitative data are presented as mean ± standard deviation. Pair-wised Student *t*-test was used to compare the differences between ES treated and control groups, with a p value < 0.05 considering statistically significant.

## Supplementary Information

**Additional file 1**:** Movie S1**. Output from the bandage when wrapped around a mouse.

**Additional file 2**:** Figure S1**. Energy-dispersive X-ray spectroscopy (EDS) spectrum of nanostructured PTFE surface.** Figure S2**. Operation of nanogenerator on the bandage.** Figure S3**. Wearable NG ES increases the healing rate of excisional wounds in nude mice.** Figure S4**. NG-driven ES modulates cells that are critical for wound healing.** Figure S5**. Thickness of new epidermis at the wound site in the control (CTL) and NG-ES treated (ES) groups.

## Data Availability

All data generated or analyzed during this study are included in this published article and its supplementary information files.

## Abbreviations

AC: Alternative current
Al: Aluminum
Au: Gold
CD31: Cluster of differentiation 31
CD68: Cluster of differentiation 68
ES: Electrostimulation
HD: High definition
H&E: Hematoxylin and eosin
ICP: Inductively coupled plasma
IHC: Immunohistochemistry
K15: Keratin 15
Ly6G: Lymphocyte antigen 6 complex locus G6D
MTT: The 3-(4,5-dimethylthiazol-2-yl)-2,5-diphenyl-2*H*-tetrazolium bromide
NG: Nanogenerator
NG-ES: Nanogenerator-driven ES
PET: Polyethylene terephthalate
PDMS: Polydimethylsiloxane
PTFE: Polytetrafluoroethylene
TGF-β: Transforming growth factor beta
