# Supplementary material for: Accelerated complete human skin architecture restoration after wounding by nanogenerator-driven electrostimulation
Source: J Nanobiotechnology. 2021 Sep 20;19:280. doi: 10.1186/s12951-021-01036-7 (PMC8454068; doi:10.1186/s12951-021-01036-7)
Supplement: Supplementary file 2 — Additional file 2: Figure S1. Energy-dispersive X-ray spectroscopy (EDS) spectrum of nanostructured PTFE surface. Figure S2. Operation of nanogenerator on the bandage. Figure S3. Wearable NG ES increases the healing rate of excisional wounds in nude mice. Figure S4. NG-driven ES modulates cells that are critical for wound healing. Figure S5. Thickness of new epidermis at the wound site in the control (CTL) and NG-ES treated (ES) groups. [file 12951_2021_1036_MOESM2_ESM.docx]

Additional file 1

Accelerated Complete Human Skin Architecture Restoration after Wounding by Nanogenerator-Driven Electrostimulation

Aiping Liu, Yin Long, Jun Li, Long Gu, Aos Karim, Xudong Wang*, Angela L.F. Gibson*


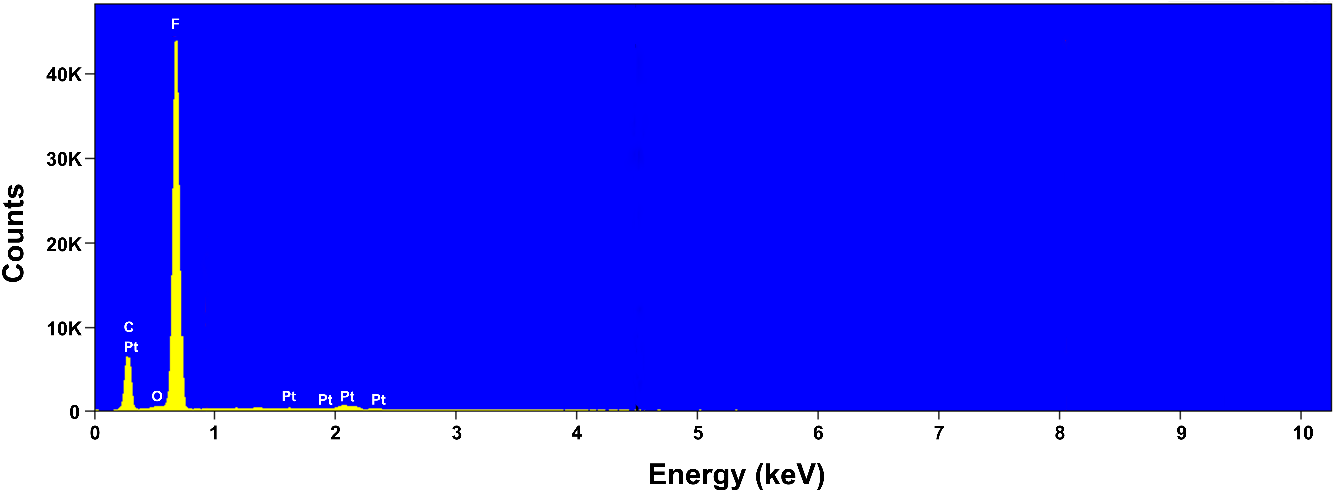


**Figure S1. Energy-dispersive X-ray spectroscopy (EDS) spectrum of nanostructured PTFE surface.**


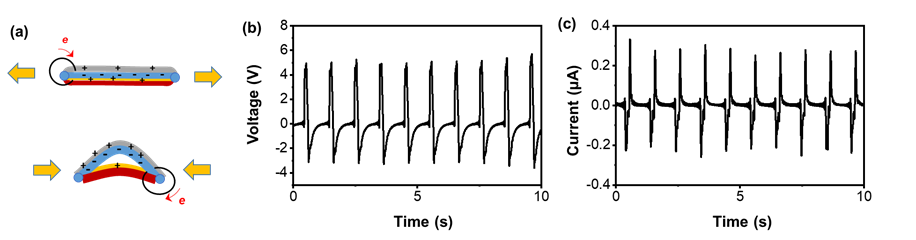


**Figure S2.** **Operation of nanogenerator on the bandage**. **(a)** Schematic operation mechanism of the triboelectric nanogenerator under straining and releasing. **(b)** Voltage and **(c)** current output of the nanogenerator under computer-controlled actuations.

**
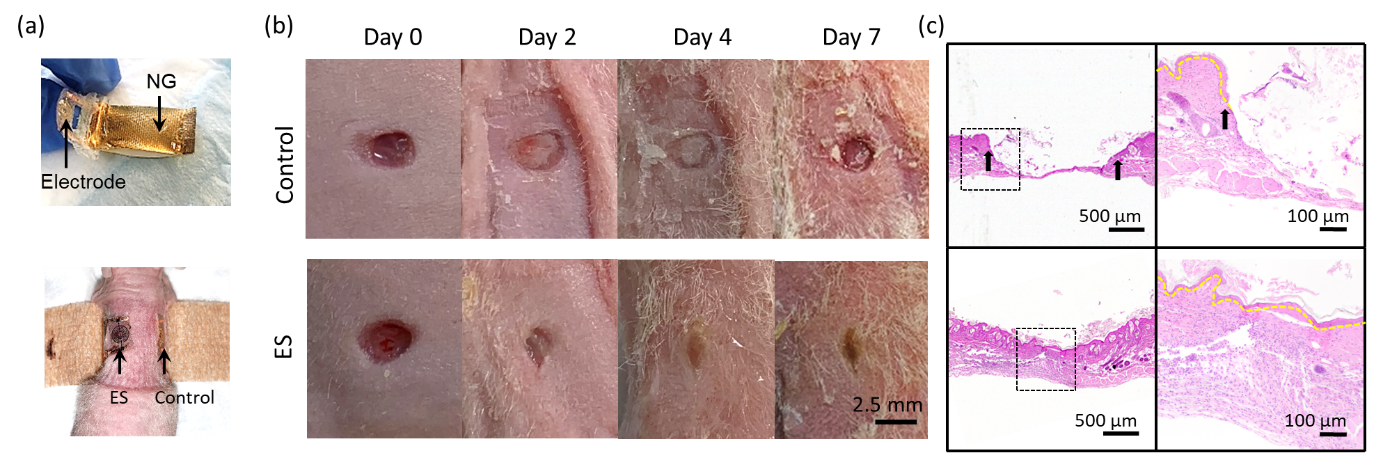
Figure S3.** **Wearable NG ES increases the healing rate of excisional wounds in nude mice. (a)** The NG-ES device on mice, **(b)** Gross photograph of wound healing after 2, 4, 7 days with or without ES treatment, and **(c)** H&E stain demonstrating complete re-epithelialization (denoted by yellow dotted line) in ES treated wounds. N= 3 per group. Arrows indicate the gap in the leading edges of neo-epidermis in non-treated wound.


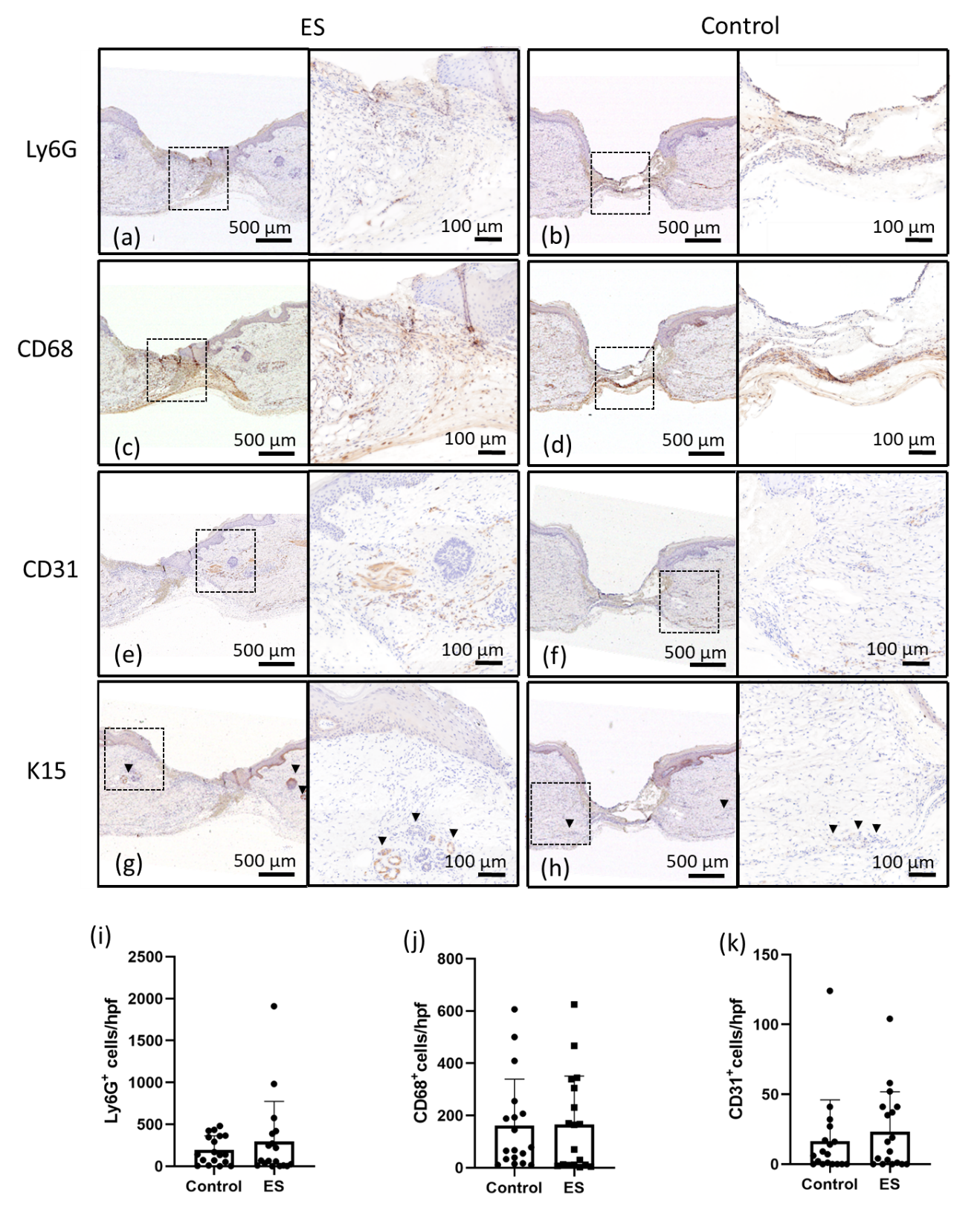


**Figure S4.** **NG-driven ES modulates cells that are critical for wound healing.** After 4 days with and without ES treatment **(a, b)** neutrophils, identified as brown Ly6G positive cells, **(c, d)** macrophages, identified as brown CD68 positive cells, **(e, f)** endothelial cells, identified as brown CD31 positively cells, and **(g, h)** epidermal progenitor cells, identified as brown K15 positive cells in the human skin grafted on mice. Arrowheads indicate eccrine glands in the K15 stained samples. There were no visible hair follicles identified on these sections. Scale bar = 500 µm low magnification; 100 µm for high-magnification insets. **(i, j, k)** Semi-quantification of Ly6G, CD68 and CD31 positive cells in 6 regions of interests at 400X magnification in the wound beds with and without 4 days of NG-ES treatment (n = 3 per group).


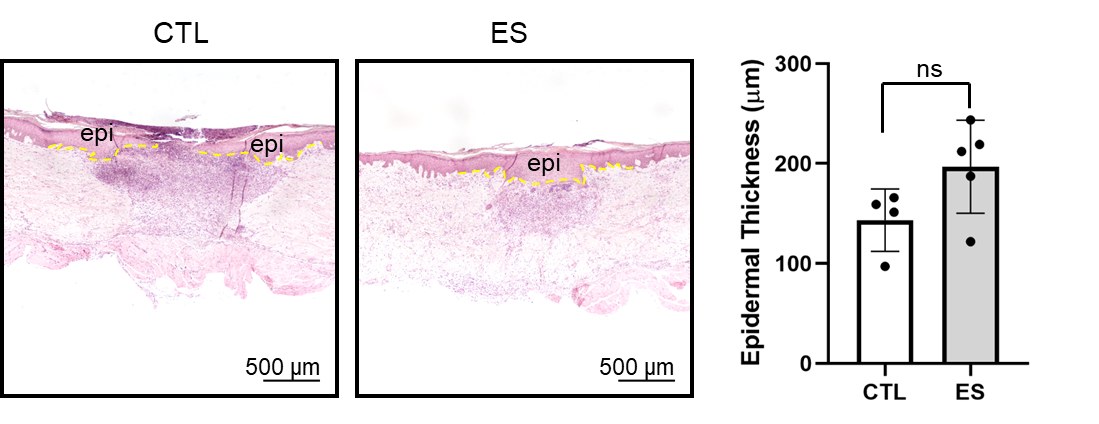


**Figure S5**. **Thickness of new epidermis at the wound site in the control (CTL) and NG-ES treated (ES) groups**. Epidermis (epi) at the wound site is delineated with a yellow dotted line from dermis. ns, non-significant.

**Movie S1. Output from the bandage when wrapped around a mouse.**
